# *Dialister pneumosintes* and aortic graft infection – a case report

**DOI:** 10.1186/s12879-023-08584-3

**Published:** 2023-09-19

**Authors:** Rachel Patel, Debra S. T. Chong, Alison J. Guy, Matthew Kennedy

**Affiliations:** 1 Royal Devon University Healthcare NHS Foundation Trust, Exeter, UK; 2 University of Exeter, Exeter, UK

**Keywords:** *Dialister pneumosintes*, Anaerobe, Aortic graft infection, MAGIC criteria, Aortoenteric fistula, Oral hygiene

## Abstract

**Background:**

*Dialister pneumosintes* is an anaerobic, Gram negative bacillus, found in the human oral cavity and associated with periodontitis. It has also been isolated from gastric mucosa and stool samples. Recent case reports implicate *D. pneumosintes* in local infection such as dental root canals, sinusitis, Lemierres syndrome and brain abscesses, as well as distal infections of the liver and lung through haematogenous spread.

**Case presentation:**

We present a novel case of aortic graft infection and aortoenteric fistula (AEF) in a 75 year old Caucasian male, associated with *D. pneumosintes* bacteraemia. Microbiological evaluation of septic emboli in the lower limbs revealed other gastrointestinal flora. This suggests either AEF leading to graft infection and subsequent distal emboli and bacteraemia, or a dental origin of infection which seeded to the graft, resulting in AEF and systemic infection. To our knowledge this is the first report of *D. pneumosintes* associated aortic graft infection. The patient underwent surgical explantation, oversew of the aorta and placement of extra-anatomical bypass graft in conjunction with antimicrobial therapy, making a good recovery with discharge home after a 35-day hospital admission.

**Conclusion:**

We report a case of *Dialister pneumosintes* bacteraemia associated with aortic graft infection. To our knowledge, vascular graft-associated infection with *D*. *pneumosintes* has not been reported before.

## Introduction

Abdominal aortic aneurysm is a focal dilatation of the aorta. Increase in diameter is associated with life threatening risk of rupture, which necessities emergency repair. Subsequently, modern healthcare providers have implemented screening programmes for abdominal aortic aneurysms and as a consequence, more aortic aneurysms are being repaired electively. There are two main techniques used for repair which both use vascular grafts – open surgery or endovascular aortic aneurysm repair (EVAR). Aortic graft infection is a serious complication following aneurysm repair. Timing of infection presentation has been used to suggest the likely microbiological flora responsible. “Early” infections (<4 months post-operatively) are more likely to involve virulent organisms introduced at the time of surgery (e.g. *Staphylococcus aureus*); conversely, “late” infection (>4 months post-operatively) may involve more indolent pathogens such as skin commensals [1]. However, AEF can be a source of infection at any time and are frequently polymicrobial involving GI bacteria and fungi [2]. Dental infections, which are commonly implicated in infective endocarditis, which is not a dissimilar infection, do not appear to have been frequently implicated in abdominal aortic graft infection. Indeed, our searches only highlighted one series that has suggested this [3].

*Dialister pneumosintes* (formally *Bacteroides pneumosintes*) is an obligatory anaerobic Gram-negative bacillus that is frequently isolated from the human buccal cavity, where is known to cause periodontitis and local infection [4, 5]. A handful of reports highlight haematogenous spread leading to severe systemic infection, including liver, lung and brain abscesses, alongside bacteraemia [6–15]. It has not been previously implicated in vascular infection, although the paucity of current literature may reflect difficulties in isolating the bacteria from traditional culture, with reports commonly suggesting identification required 16s PCR [6–12].

## Case report

A 75 year old independent Caucasian male presented to hospital with three weeks of pyrexia and lethargy, alongside progressive bilateral tender palpable masses to his lower limbs. He was normally well and was physically active prior to this illness. His past medical history was significant for an elective AAA repair 11 months previously, which has been complicated by an upper gastrointestinal bleed post-operatively. He was investigated for this with upper and lower endoscopy which showed gastritis and haemorrhoids only. His regular medication included a statin, clopidogrel and omeprazole.

Upon review, he was pyrexial and tachycardic - HR 124, temperature 38.1^o^C, BP 138/80, RR 18, SpO2 99% OA. Examination revealed slow gait due to leg pain and hot tender lumps in the lower limbs. There were no peripheral stigmata of infective endocarditis. Chest and abdominal examination was unremarkable. GCS 15/15. Clerking bloods showed high inflammatory markers – WCC 24.8, neutrophils 21.27, CRP 166. Empiric antimicrobial treatment was intravenous Piperacillin/Tazobactam and intravenous vancomycin.

**Fig. 1:**
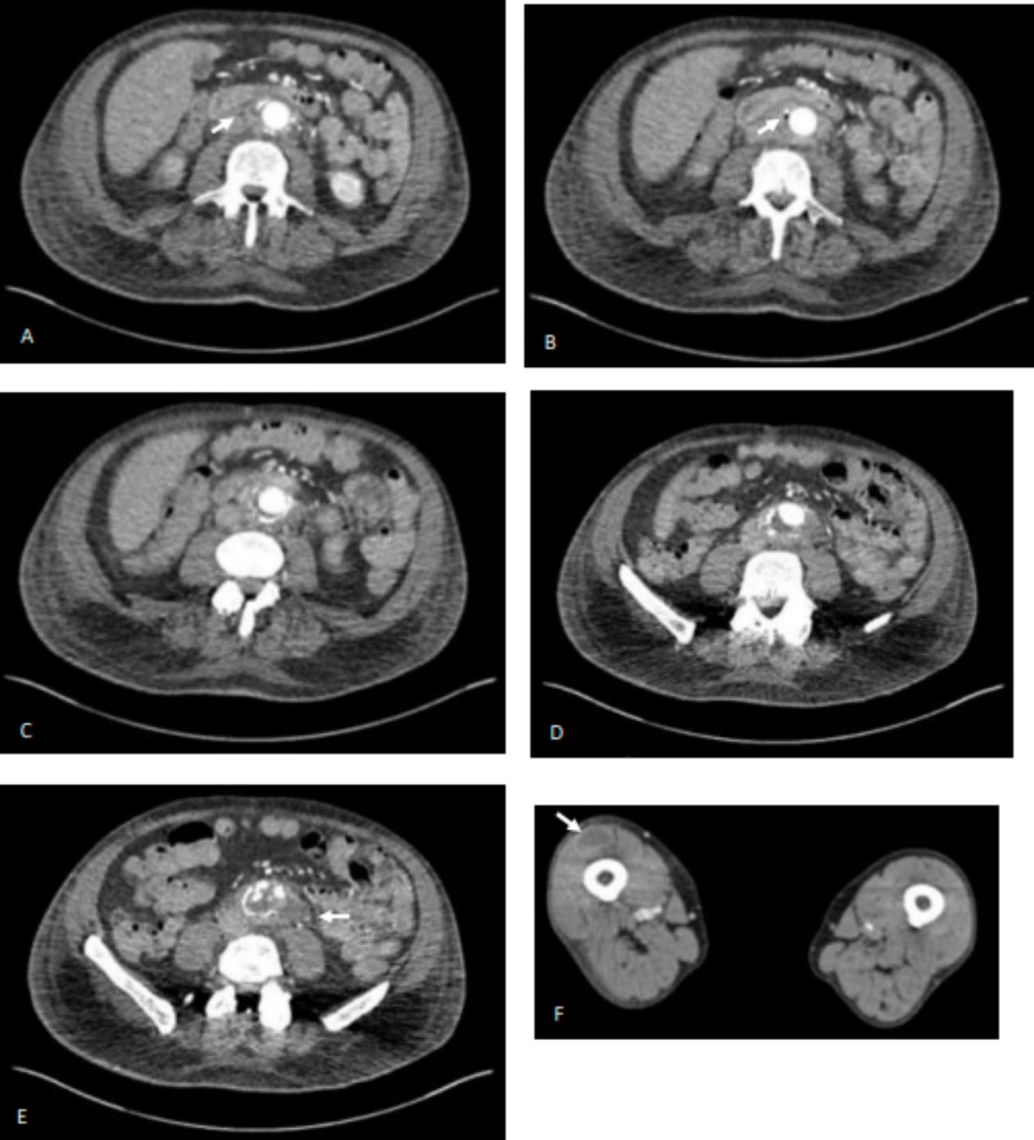
**A-E** represent caudal to rostal axial images from admission CT - aortic graft infection confirmed with locules of gas and multiple collections around the abdominal aorta. **F** shows right vastus lateralis collection

CT angiogram of the aorta confirmed aortic graft infection with distal emboli: locules of gas and multiple small collections surrounding the aortic graft were noted, as well as soft tissue collections in left gluteus minimus, right inferior gluteus maximus, right vastus lateralis, right tibialis anterior, right posterior calf, and left soleus (Fig. 1).

Of the five sets of blood cultures drawn on the day of admission, only the anaerobic bottle of one set flagged positive at 37 h. All blood cultures were incubated in a dedicated Becton Dickinson BACTEC FX unit (Worthing, United Kingdom). Gram stain revealed a combination of Gram positive cocci in chains and small Gram negative cocci This was subcultured to blood, chocolate and Neomycin fastidious anaerobe agar. The anaerobic plate was incubated in a dedicated anaerobic cabinet – (Whitley A45 Workstation Bingley, United Kingdom) at 35-37 degrees centigrade as per the conditions in the UK SMI [13]. All plates were examined every 24 h. Growth was only seen on the anaerobic plate and subsequent Bruker MALDI-TOF Biotyper Sirius (Bruker, Massachusetts, United States) identification revealed *D. pneumosintes* (Score 1.97 – Version 11 Bacteria Database). The Gram stain was reviewed by a second biomedical scientist who thought that, what was initially reported as Gram negative cocci could, infact, be Gram negative short rods, which would be in keeping with *D. pneumosintes*. Disc diffusion susceptibility plates were set up with the results shown in Table 1. Inhouse antibiotic gradient strips to determine MIC (ug/ml) were also performed with the following results:, Co-amoxiclav <0.016, Metronidazole 0.064.

**Table 1:** Disc diffusion antibiotic susceptibility data for D. pneumosintes

Antibiotic Disc	Zone Diameter (mm)
Amp	27
Pen	19
Ery	20
Clinda	30
Tet	> 35
Chlor	> 35
Lev	30
SXT	25
Vanc	6
LZD	6
Taz	> 50

Fluid aspirated from leg collections isolated other gastrointestinal flora commonly isolated from the mouth - *Streptococcus anginosus*, *Fusobacterium nucleatum*, and *Parvimonas micra*. Given these findings, a maxillofacial review was requested along with orthopantomography. Opinion was of poor dentition but not acute dental infection requiring intervention. Once antibiotic sensitivities were confirmed on all isolates, antimicrobial spectrum was narrowed with transition from Piperacillin/Tazobactam and Vancomycin to 2.4 g IV benzylpenicillin every four hours and 400 mg IV metronidazole every eight hours. Endocarditis dosing regimen of gentamicin (1 mg/kg twice daily) was added to cover for possible infective endocarditis despite repeatedly negative transthoracic echocardiograms.

Definitive aortic graft explantation was undertaken three weeks after admission. The planned operation involved graft explantation and replacement with biological graft. However, surgery was complicated by intraoperative findings of a small AEF between the redundant aneurysm sac and the duodenum. Therefore, the duodenum was repaired with an omental patch and a new axillo-bifemoral graft sited away from any local sources of infections. The fistula also prompted the addition of empiric fluconazole (800 mg loading dose, subsequently 400 mg IV once daily). The intraoperative aortic graft and sac samples isolated *Citrobacter koseri* and *Candida albicans* in keeping with AEF. Fluconazole gradient diffusion etest revealed *Candida albicans* MIC 1.5 and EUCAST antifungal breakpoints were applied to determine susceptibility [14]. *D. penumosintes, S. anginosus, F. nucleatum or P. micra* were not isolated from theatre samples, but this is not surprising as our patient received three weeks of effective anti-anaerobic antimicrobial therapy prior to culture.

Antimicrobial therapy was rationalised post operatively to IV co-amoxiclav 1.2 g TDS and caspofungin 70 mg loading dose and then 50 mg thereafter OD. Transition to oral co-amoxiclav 625 mg TDS and 400 mg fluconazole OD was recommended when clinically appropriate. A total of six weeks of co-amoxiclav was suggested post explantation, with six months of fluconazole on discharge. We are pleased to report that the patient was reviewed 6 months after discharge and had made a full recovery with no limitations to activities of daily living and good exercise tolerance.

## Discussion

*D. pneumosintes* is well established as a cause of periodonitis and gingivitis in humans but reports of infection at other sites are sparse [4]. A PUBMED search for *D. pneumosintes* case reports produced twelve results of extra-oral infection (reference 9 reports on two patients), including orbital cellulitis and sinusitis, as well as pneumonia and empyema, liver and brain abscesses [5–12, 15–17]. In seven of the twelve cases *D. pneumosintes* was isolated from blood culture, suggesting haematogenous spread with a tendency for distal abscess formation. Three report polymicrobial infection; one identified mixed anaerobic infection with *P. micra*, as isolated in our case. However, there are no previous reports of aortic graft infection, other vascular infection, or indeed infection associated with other prosthetic material. *D. pneumosintes* has been isolated from lower in the gastrointestinal tract, in gastric and colorectal mucosal biopsies, stool samples and intraabdominal drain fluid [18–21]. This case highlights a novel case of bacteraemia with concurrent aortic graft infection *with D. penumosintes*. Whether this was caused by AEF with resultant aortic graft infection and bacteraemia, or whether distal seeding from periodontal infection led to graft infection and erosion to AEF is debatable.

Identification of *D. pneumosintes* is difficult with routine culture techniques as it is a slow growing obligate anaerobe. Therefore, current publications likely reflect a small sample of the true number of *D. pneumosintes* infections. Of the twelve case reports identified, only two isolated *D. pneumosintes* using traditional culture, compared with nine that used molecular methods (16 S rRNA gene sequencing or whole genome sequencing). Molecular methods have the added benefit of increased sensitivity following exposure to antimicrobial therapy. Unfortunately, we did not have the capacity to test the surgical material from explantation, or dental samples which would have proven source, with 16 S PCR for *D. pneumosintes*.

Literature reports suggest *D. pneumosintes* may be resistant to antibiotics commonly used for polymicrobial GI infections such as fluroquinolones, vancomycin, colistin and macrolides [21–23]. Our isolate was, unsurprisingly, resistant to vancomycin and linezolid. Although all positive blood cultures in the UK will have susceptibility work up, this highlights the importance of antimicrobial susceptibility testing on severe infections, especially those caused by unfamiliar bacteria.

In 2020, the Management of Aortic Graft Infection (MAGIC) group developed a clinical/surgical, radiological and microbiological list of criteria, which are classified into major and minor, to help suspect and diagnose vascular graft infections (Table 2) [24]. Our patient fits these criteria with a CT scan 11 months after aortic graft insertion showing gas in peri-graft collections. The positive blood cultures and fevers are minor criteria. This registry provides useful management of infected aortic grafts and highlights gaps in current knowledge. Of note, our case underwent explantation of the original vascular graft with placement of an extra-anatomical prosthetic graft. In Sect. 7.2.3 of the MAGIC guidelines, re-infection rates using this technique are quotes as 0-15%, and in smaller series the rate is up to 27%. However, there is no overall consensus as to antimicrobial therapy duration, with some research advocating up to one year of antimicrobial therapy post-op. We opted for 6 weeks post-op of antibiotics and 6 months of fluconazole.

**Table 2:** The MAGIC criteria 2020 suggest vascular graft infection diagnosis with >/= 1 major criterion and any other criterion from another category [24]

	MAJOR	MINOR
**Clinical/surgical**	• Pus (on microscopy) around graft or in aneurysm sac at surgery • Open wound with exposed graft of communicating sinus • Fistula development e.g. aorto-enteric	• Localised clinical features of graft infection e.g. erythema, swelling, discharge, pain • Fever > 38oC with graft infection as most likely cause
**Radiological**	• Perigraft fluid on CT scan > 3 months after insertion • Perigraft gas on CT scan > 7 weeks after insertion • Increase in perigraft gas volume demonstrated on serial imaging	• Other findings e.g. suspicious perigraft gas/fuid; soft tissue inflammation; aneurysm expansion; pseudoaneurysm formation; focal bowel wall thickening; discitis/osteomyelitis; suspicious metabolic activity on PET CT or radiolabelled leukocyte scan
**Laboratory**	• Organisms recovered from explanted graft • Organisms recovered from an intraoperative specimen • Organisms recovered from a percutaneous, radiologically guided aspirate of perigraft fluid	• Blood culture positive, no apparent source except graft infection • Abnormally elevated inflammatory markers (e.g. CRP, white cell count) with graft infection as most likely source

To conclude, we report a case of aortic graft infection and AEF caused by mixed oral flora including *D. pneumosintes*, AEF are often difficult to diagnose and prompt recognition is vital to reduce the risk of devastating consequences. Polymicrobial abscess without other explanation in a patient with a vascular graft lead to investigation for AEF. In this case the absence of overt acute dental infection suggests that fistula may have led to local infection with subsequent bacteraemia and distal emboli. *D. pneumosintes* alone was isolated from anaerobic blood culture by traditional culture methods, prior to antimicrobial exposure; S. *anginosus*, *F. nucleatum* and *P. micra* were isolated from distal septic emboli in the lower limbs. Suggestions for future research into microbiological association with aortic graft infection include patient’s dental hygiene and buccal flora, as well as length of antimicrobial therapy following explantation and replacement of extra-anatomical prosthetic graft.

## Data Availability

Data sharing is not applicable to this article as no datasets were generated or analysed during the current study.
